# Enhancing renewable energy certificate transactions through reinforcement learning and smart contracts integration

**DOI:** 10.1038/s41598-024-60527-3

**Published:** 2024-05-12

**Authors:** Qingsu He, Jinsong Wang, Ruijie Shi, Yifan He, Muqing Wu

**Affiliations:** 1 State Grid Digital Technology Holdings Co., Ltd. (State Grid Xiongan Financial Technology Group Co., Ltd.), Beijing, 100010 China; 2 Case Western Reserve University Cleveland, Cleveland, 44106 USA; 3 University of California, Santa Cruz, 95064 USA; 4 College of Information and Communication Engineering, Beijing University of Posts and Telecommunications, Beijing, 100876 China

**Keywords:** Green certificate, Nash Q-learning, Smartcontract, Renewable energy, Carbon asset, Energy science and technology, Renewable energy, Electrical and electronic engineering

## Abstract

Given the complexity of issuing, verifying, and trading green power certificates in China, along with the challenges posed by policy changes, ensuring that China’s green certificate market trading system receives proper mechanisms and technical support is crucial. This study presents a green power certificate trading (GC-TS) architecture based on an equilibrium strategy, which enhances the quoting efficiency and multi-party collaboration capability of green certificate trading by introducing Q-learning, smart contracts, and effectively integrating a multi-agent trading Nash strategy. Firstly, we integrate green certificate trading with electricity and carbon asset trading, constructing pricing strategies for the green certificate, carbon, and electricity trading markets; secondly, we design a certificate-electricity-carbon efficiency model based on ensuring the consistency of green certificates, green electricity, and carbon markets; then, to achieve diversified green certificate trading, we establish a multi-agent reinforcement learning game equilibrium model. Additionally, we propose an integrated Nash Q-learning offer with a smart contract dynamic trading joint clearing mechanism. Experiments show that trading prices have increased by 20%, and the transaction success rate by 30 times, with an analysis of trading performance from groups of 3, 5, 7, and 9 trading agents exhibiting high consistency and redundancy. Compared with models integrating smart contracts, it possesses a higher convergence efficiency of trading quotes.

## Introduction

Amid rising global attention to climate change, the development and use of renewable energy has become crucial for reducing greenhouse gas emissions and achieving sustainable development goals. In China, the world’s largest energy consumer and carbon emitter, developing zero-carbon energy and promoting renewable energy consumption through green certificates (GCs) are essential for meeting increasing energy needs and striving to achieve carbon neutrality by 2060. Consequently, China has introduced measures such as the Renewable Energy Portfolio Standards (RPS)^1,2^ and Tradable Green Electricity Certificates (GEC) systems to encourage a shift towards sustainable energy production and consumption through market incentives.

The experiences of the United States, the United Kingdom, Italy, Norway, and Sweden provide valuable lessons for China, demonstrating the effectiveness of market mechanisms in promoting renewable energy development.

Due to varying policies and green certificate initiation times across countries, international green certificate systems also face limitations, especially in terms of policy impact and market adaptability, such as price volatility and regulatory challenges that could undermine investor confidence and the economic viability of renewable projects. To enhance the efficiency and adaptability of the green certificate market, innovative methods are being explored, including the use of blockchain technology and the development of green financial products, as well as international cooperation.

Moreover, China’s green certificate market still confronts numerous challenges^3^, including the complexities of issuance, verification, and trading, and the impact of policy changes. The current lack of a market-based trading system hampers the interconnectivity of electricity trading, carbon emission rights trading, and the realization of the environmental attributes of green certificates. The absence of an effective pricing mechanism and sufficient market incentives has led to low transaction volumes, highlighting the urgent need to improve the green certificate trading framework, increase transaction efficiency, and transparency to promote renewable energy development^4^.

In light of these issues, this study proposes a zero-carbon energy green certificate trading system (GC-TS) architecture based on a balanced strategy, integrating Q-learning, smart contracts, and multi-agent Nash strategies. This aims to address current market problems, enhance transaction efficiency, and foster collaboration, making significant contributions to sustainable development goals in China and globally. Through this comprehensive strategy, the goal is to push the green certificate market towards higher efficiency, transparency, and intelligence, ensuring the harmonious coexistence of economic growth and environmental sustainability.The main contributions are:

1. To establish a multi-intelligence reinforcement learning game equilibrium model with the goal of diversified GC trading;
2. Introducing smart contracts to optimize the game equilibrium efficiency of multi-intelligents participating in trading, and establishing a trading system framework.

### Literature review

The global transition to sustainable energy sources necessitates the development of mechanisms like green certificates (GCs) to incentivize renewable energy production. Scholars from China, Europe, America, and other regions have extensively researched and explored issues related to the market mechanisms and models of GCs^5–19^, technological innovations including blockchain and artificial intelligence platform technologies^20–37^, policies and economic strategies and market changes^10,19, 38–45^.

Researchers worldwide, especially in China, Europe, and the United States, have thoroughly explored the rules of the green certificate market^5–8^, circulation methods^9^, price simulations^10^, and market models^11,12^. Through trading games^13–15^, equilibrium models^16^, the integration of electricity markets with green certificate markets^17–19^, the coupling of green certificates with carbon emissions^46,47^, market combinations^48^, the impact of carbon pricing^49^, and the effect of electricity load^50^, scholars aim to understand and optimize the dynamics and efficiency of the GC market.

With the evolution and development of information processing technology, utilizing blockchain and artificial intelligence in the pricing system of GCs, trading strategies, and optimizing transaction efficiency has become an important means in the design of new generation trading technology frameworks. The application of blockchain technology^20–23, 51^, smart contracts^25,26^, transaction information processing^27–30^, and platform design^31,32^ has offered new possibilities for GC trading. These technological innovations not only enhance the transparency and efficiency of transactions but also promote the security and reliability of the market.

Research also involves the policies and economic strategies of GCs^38,46^, especially analyzing how renewable energy tariff surcharge subsidies, subsidy settlement cycles, delay cycles, and consumer preferences affect the sales price of GCs. These strategies aim to improve the market price advantage of GCs^52^, thereby promoting the consumption of renewable energy.

In the GC market, the strength of the herding effect determines the average compliance cost level, and the strategic behavior of market participants mainly affects the stability of the GC price, with greater price volatility leading to slower convergence to the equilibrium price^39^. To address the regional imbalance of energy supply and demand in China, literature^53^ has established a cross-regional GC futures model. Additionally, research has used game models^40–44^, self-conclusive and variational particle swarm optimization algorithms^45^, and other methods to assess the synergy and incentives of electricity^54^, GPMs, and the GC market on the decision-making behaviors of non-renewable energy generation companies^55,56^, GPMs, and electricity purchasers.

The application of reinforcement learning and Q-learning in financial market forecasting^33^, learning trading rules for specific financial assets^34^, and improving financial trading decisions^35^ offers a new perspective for GC trading strategies. Particularly, deep Q-learning in the algorithmic trading system for the commodity futures market^36^ and the design of a supply chain carbon allowance allocation auction based on multi-agent modeling and Q-learning^37,57,58^ demonstrate the potential of AI technology in energy management and GC trading.

Despite the challenges posed by the complexity of issuing, verifying, and trading GCs, as well as policy changes, the development of the GC market can be effectively enhanced by integrating technological innovations and advanced trading strategies, such as reinforcement learning and smart contracts. Future research needs to focus on further integrating these technologies and strategies, as well as addressing challenges specific to the Chinese market, to contribute to the healthy development of the global GC market.

## Green certificate framework: Methods and lifecycle

**Figure 1:**
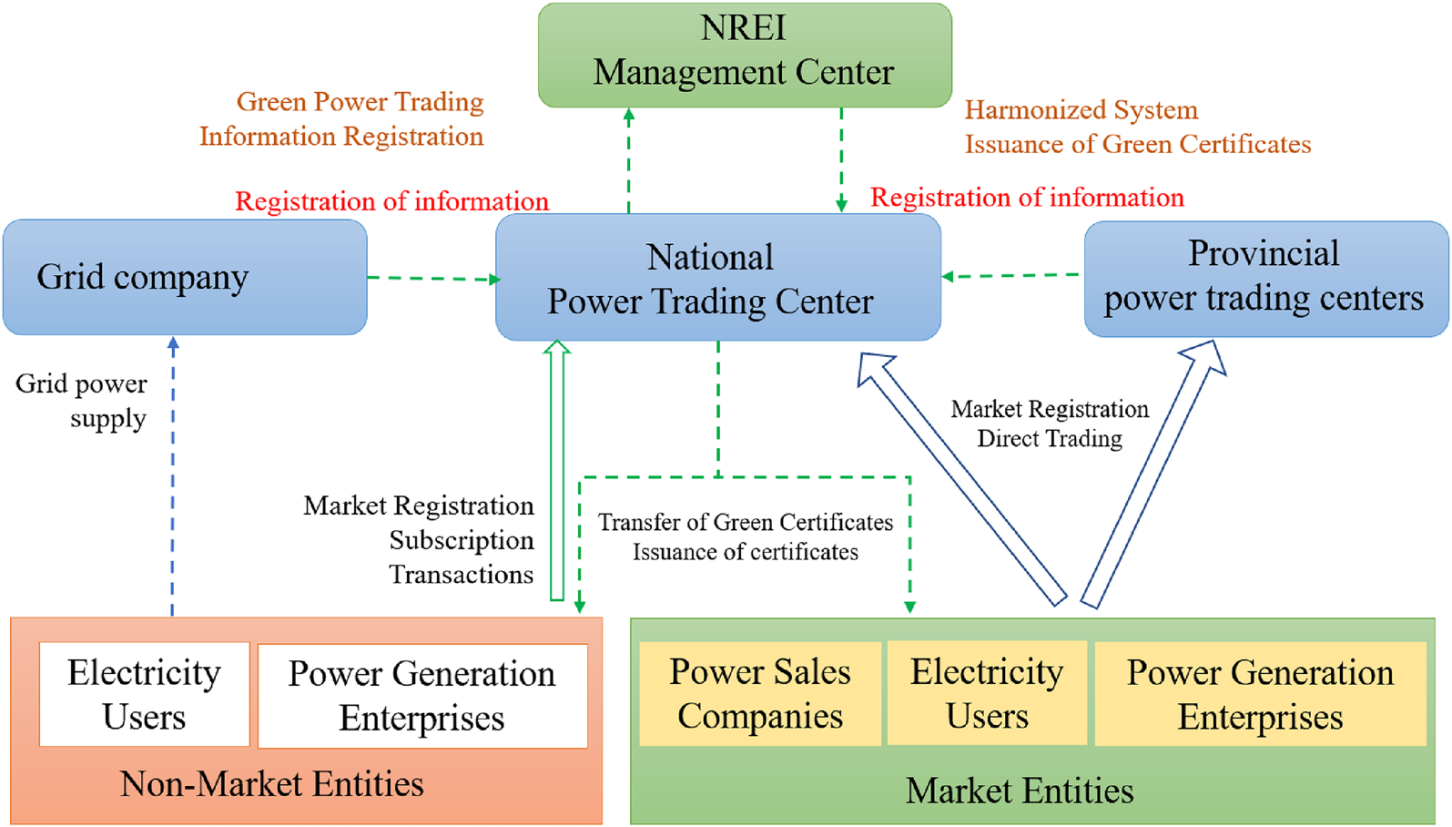
Framework for green certificates to accompany green power trading in China.

**Figure 2:**
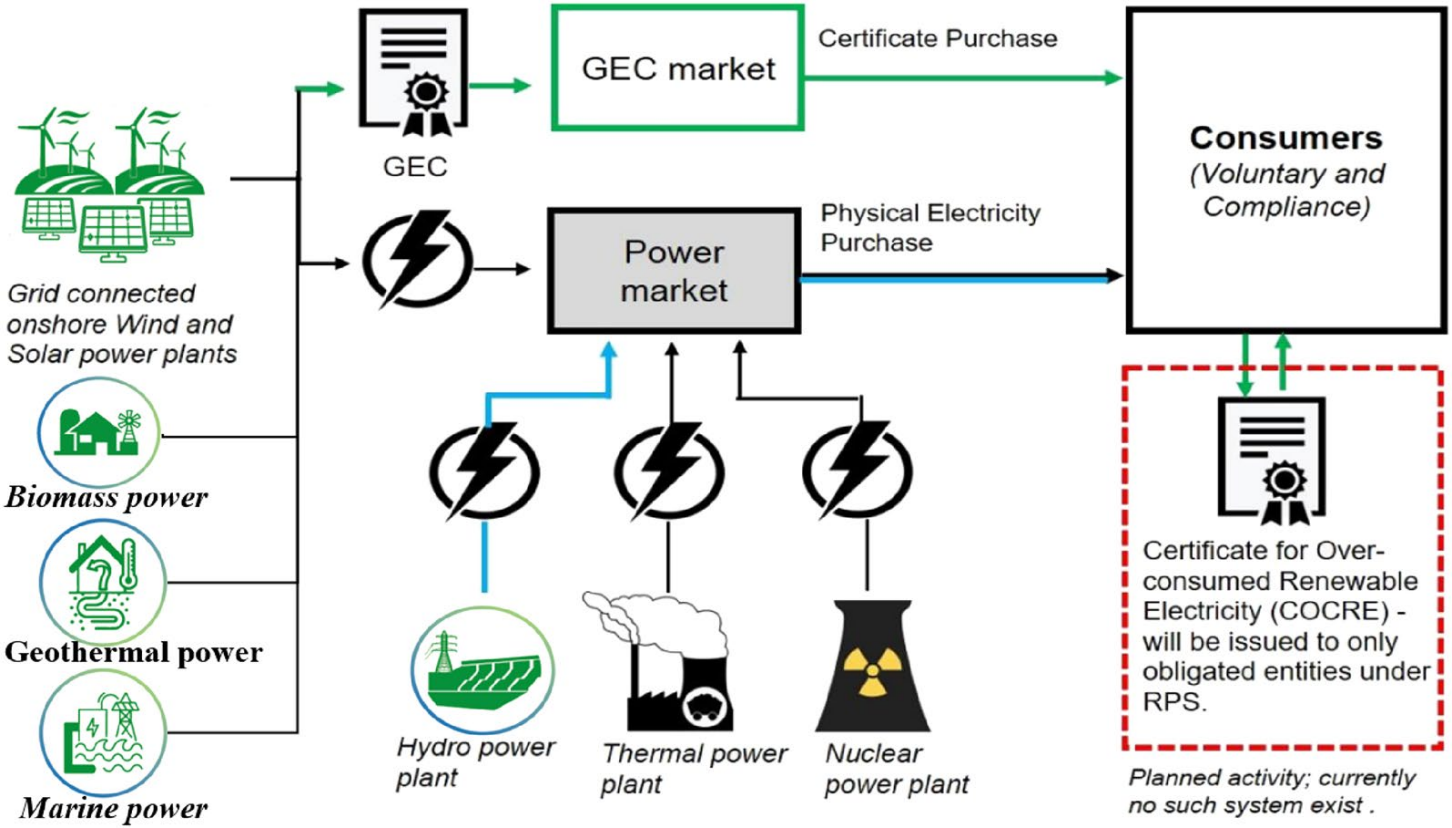
Schematic diagram of the green certificate and green electricity separation trading method.

The trading model for GCs primarily comprises two components: GCs bundled with green power (as Fig.  1) and standalone GCs (as Fig.  2).

The trading of Green Certificates (GCs) alongside green power represents a dual model that integrates both “certificates and power.” This model enhances the traceability of green power throughout its entire lifecycle and aligns the value of green power with environmental benefits. In the process of green power transaction settlements, the power trading center allocates GCs based on various data points that have been mutually agreed upon by the involved parties. The established framework, as depicted in Fig.  1, ensures a harmonized flow of green power and the corresponding GCs, thereby simplifying trading processes and strengthening market participation.

Independent trading certificates allow renewable energy producers to trade on a voluntary subscription platform, where buyers select GCs through the interface and complete their subscription via negotiation, listing, or auction. Although this method overcomes geographical limitations of electricity transmission, it faces challenges: the platform’s inability to integrate electricity market data makes it difficult to bridge the gap between “certificates and electricity,” and there’s a lack of a quantified relationship between GCs and carbon credits for achieving net-zero carbon. NREI will issue a certificate (One Ps./MWh) based on a certain proportion of green power traded, forming a tradable GC. Generally speaking, grid companies, power sales companies, power users, and carbon emission enterprises (such as fossil energy power plants) constitute the main body of the quota. QEs fulfill their social responsibilities of green environmental protection, carbon reduction, and low carbon by purchasing GCs. GC life cycle is divided into three stages: issuance, Transactions, and verification.

## Revenue model: based on green power manufacturers and quota enterprises

GPMs sell GCs in the trading market to subsidize their initial investment costs. The price of the GC will be affected by time, region, supply and demand tension, and other factors. The total revenue of the GPM settlement cycle $u_{gpm}$ includes electricity sales revenue $I_{rep}$, government subsidy income $I_{(rep,subs)}$, and GC transaction revenue $I_{gc}$.

1 $$\begin{aligned} \begin{aligned} u_{gpm} = I_{ep}+I_{(ep,subs)}+I_{gc}-C_{ep} =I_{(ep,subs)}+\sum _{t=1}^{T}{(\lambda ^{ep}_t*Q^{ep}_t+\lambda ^{gc}_t*Q^{gc}_t)}-C^{ep}. \end{aligned} \end{aligned}$$

where $\lambda ^{gc}$ is the price of GCs, $Q^{gc}$is the quantity of GCs,$\lambda ^{ep}$ is the on-grid price of renewable energy power generation, and $Q^{ep}$ is the electricity generation of RE,$C^{ep}$ is the cost of generation of the RE. It is assumed that this work does not consider the impact of government subsidy income $I_{(ep,subs)}$ on total income. Equation (1) can be expressed as:

2 $$\begin{aligned} u_{gpm}=\sum _{p\in {P}}\sum _{t\in {T}}{(\lambda _{p,t}^{ep}*Q_{p,t}^{ep}+\lambda _{p,t}^{gc}*Q_{p,t}^{gc})}-C^{ep} \end{aligned}$$

where $\lambda _{p,t}^{ep}$ and $Q_{p,t}^{ep}$ are the online trading electricity price and trading electricity quantity of GPMs in times(*t*) ,$\lambda _{p,t}^{gc}$ ,$Q_{p,t}^{gc}$ are the transaction price and transaction quantity of GCs of GPMs in times(*t*).

3 $$\begin{aligned} C^{ep}=\sum _{p\in {P}}\sum _{t\in {T}}{(m_{ic,p}*\hbar _{p,t}*\lambda _{p,t}^{cost})} \end{aligned}$$

where $\lambda _{p,t}^{cost}$ is the unit cost of electricity generated by renewable energy,$m_{ic,p}$ is the installed capacity of renewable energy, and $\hbar _{p,t}$ is the monthly utilization hours of renewable energy generation. Assume that all renewable energy traded electricity will receive GC, satisfying $m_{ic,p}*h_{p,t}=Q_{p,t}^{ep}$. Equation (2) can be simplified as follows:

4 $$\begin{aligned} \left\{ \begin{array}{l} u_{gpm}=\sum _{p\in {P}}\sum _{t\in {T}}{((\lambda _{p,t}^{ep}-\lambda _{p,t}^{cost})*Q_{p,t}^{ep}+\lambda _{p,t}^{gc}*Q_{p,t}^{gc})} \\ s.t.Q_{p}^{ep} \ge Q_p^{c},\lambda _p^{ep} \ge \lambda _p^{cost} \end{array} \right. \end{aligned}$$

without considering other commercial values of GC in Eq. (4), we consider that its market transaction price can be determined by green power cost, revenue balance and opportunity cost.

The utility function of the QE corresponds to its cost function, and the model is as follows:

5 $$\begin{aligned} \left\{ \begin{array}{l} u_{qe}= \sum _{p\in {P}}\sum _{t\in {T}}{(\gamma _{p,t}*Q_{p,t}^{ep} + \lambda _{p,t}^{co_2} \varphi (Q_{p,t}^{gc})-\lambda _{p,t}^{gc}*Q_{p,t}^{gc})} \\ \varphi (Q_{p,t}^{gc}) = \omega Q_{p,t}^{gc}+\varPi _{p,t}^{(co_2,qe)} \end{array} \right. \end{aligned}$$

where $u_{qe}$ is the total cost of the QE, $\lambda ^{ep}$ is the on-grid electricity price (the fixed electricity price model will be used in this work),$\lambda ^{gc}$ is the GC transaction price of the QE,$Q^{gc}$ is the GC transaction volume of the QE. $\lambda _{p,t}^{co_2}$ is the price of carbon.$\gamma _{p,t}$ is the ratio of income generated from the production and operation of enterprises using renewable energy (We take the reciprocal of carbon emission intensity of QE as the calculated value of $\gamma$ in this work).

To reflect the characteristics of the seller’s market in the function, this work supplements the function $\varphi (Q_{p,t}^{gc})$ as a component in the cost function of the QE, which represents the ability of a QE to independently complete a certain amount of quota.It is also a functional relationship between the actual GC transaction volume and a certain percentage of the quota. In this work, the quota completion capability function is a constraint rather than a real cost for the QEs. QE needs to consider whether it can meet the quota requirements when bargaining with GPMs and adjusting the transaction volume strategy. $\omega$ is the carbon asset conversion rate corresponding to the GC (and the quota rate of the QE). Where $\varPi _{p,t}^{(co_2,qe)}$ is the actual GC trading volume of the enterprise that offsets the carbon quota quantity.We ignore the impact of income change trend. In addition, the price of GCs and the price of carbon assets meet a certain linear relationship (the coefficient is $\eta$ ): $\varPi _{p,t}^{(co_2,qe)} =\gamma _{p,t} * \eta * Q_{p,t}^{gc}$ So transformed Eq. (5) as follows:

6 $$\begin{aligned} \left\{ \begin{array}{l} u_{qe}= \sum _{p\in {P}}\sum _{t\in {T}}{(\gamma _{p,t}*Q_{p,t}^{ep} + \lambda _{p,t}^{co_2} \varphi (Q_{p,t}^{gc})-\lambda _{p,t}^{gc}*Q_{p,t}^{gc})} \\ \varphi (Q_{p,t}^{gc}) = \omega Q_{p,t}^{gc}+\gamma _{p,t} * \eta * Q_{p,t}^{gc} \end{array} \right. \end{aligned}$$

Equation (6) establishes the correlation between carbon allowances, green certificates, and carbon intensity. Equations (4), (6) provide quantitative relationships for game equilibrium, trading offer optimization, and reinforcement learning convergence.

## Algorithms combining game strategies: Q-learning and smart contracts

### Game and green certificates

We define the multi-agent collaborative trading game, $G = \langle P, S, U \rangle$. Where *P* is the set of QEs, GPMs of GCs, $P = {1, 2,..., n }$; *S* is the set of possible strategies to be executed by the transaction; $S_i$ is the action strategy of each buyer(seller) in the team, and each buyer(seller) makes the corresponding action according to the current surroundings and the environment of other participants. The strategy of each Agent can be formalized as $( A_t^1, A_t^2,..., A_t^n )$, and *U* is the payoff function, which denotes the gain or loss after executing the strategy.

In dynamic and complex environments, the information acquired by agent in a multi-*Agent* system may be complete or incomplete. Let the environment in which a multi-*Agent* system is located be *X*, and $X_t$ denotes the environment in which the multi-*Agent* system is located at moment *t*. Let the set of agent observable environmental states be $S_t$, $S_t^i = f (E_t)$, and $S_t =(S_t^1,S_t^2,...,S_t^n )$ be the joint observation in the system at moment *t*, denoted by $S =\prod _{i \in N}S_i$. Let the set of agent actions be *A*, $A^i$ is used to denote the set of actions of agent *i*, the set of actions of agent is denoted by *A*( $A =\prod _{i \in N}A_i$), and from the impact on the environment of the actions taken by each agent at the moment *t* of the observed environment $A_t^i \in A_i$, the joint action between multiple participants $( A_t^1, A_t^2,..., A_t^n )$ will also affect the state of the environment in which it is currently located.

Let the state transfer function be *T*, $T_t: S \times A \rightarrow S$, denoting the possible impact on the environment through collaboration between a trader and other transactions in a particular environment.

Let the Agent payoff function be *U*, $U_i = S \times A \rightarrow U$, denoting the payoffs after the actions taken by agent *i* to accomplish a task in a multi-agent system. Agent’s goal set $G = { G1, G2,..., Gn}$, $G_i$ denotes the goal of each agent in the multi-agent system, which can usually be expressed by using the payment function *U*, and there may be multiple relationships between the goals of each agent *i*: when the goals are consistent, the completion of the goals between the Agents is mutually reinforcing; when the goals conflict, there is a conflict of interest resources. If there exists a joint action $a^* \in S$ that satisfies the conditions: $\forall I \in P$, $\forall a_i \in S, U (a^*_i, a^*_{i-1}) \ge U (a_i,a_{i-1})$, then $a^*$ is said to be a Nash equilibrium of the game *G*.

According to Eqs. (4), (6), the GMP revenue function is a quadratic function of the green power transaction and GC market price, with a coefficient of not less than zero and a “convex” property.Therefore, the game model considered has Nash equilibrium.Through the process of establishing the above mathematical model,the GC trading price ($\lambda ^{ep},\lambda ^{gc}$) is established based on the transformation of the interests of the participating subjects. The trading subjects derive the optimal strategy based on the combination of variables( $Q_{i,t}^{ep}$,$Q_{i,t}^{gc}$,$\varPi _{i,t}^{co_{2},qe}$) and adjustment coefficients ($\gamma _{i,t},\eta ,\omega$), and thus determine the Nash equilibrium point.Considering the introduction of non-convex characteristics of decision variables in equation. The traditional solution algorithm has certain difficulties, so this paper adopts the Therefore, this paper adopts a multi-agent Nash-Q reinforcement learning algorithm and Smart matching of bidding under smart contracts.

### Q-learning-based strategy analysis and green certificate trading process

Q-learning have been heavily researched in finance, especially in stock trading, and in the energy field focusing on energy management. Given the attributes of GCs and the market participants in the transaction subject, in addition to the different liquidity, the mode of its transaction can be referred to. So,we combine multi-agent reinforcement learning with game theory and construct a framework for applying multi-participant bidding games in GC markets using multi-agent Nash-Q reinforcement learning. Agents try out rewards or punishments given by the environment during the learning process and gradually develop expectations about incentives to formulate reward-maximizing strategies. We adopt the Q-learning algorithm in reinforcement learning, which involves four kinds of parts: (1) Q-table, $Q(s,a^1,...,a^n)$ is the cumulative value of executing *a* action in state *s*, (2) selecting action *a*, (3) making action and environment feedback, 4) environment update. During its process $Agent_i$ observes the surrounding environment and executes the actions in the action strategy set. At time *t*, $Agent_i$ acts $a_i$, while feedback gain $R(S_t, a_i)$, updates the Q-value table, and repeats the above process until the end of the task. The value of $Q(S_t, a_i)$ can be expressed as: $Q(S_t,a_i)=R(S_t,a_i) +\beta max Q(S_t+1,a_i+1)$, where *a* is an action in the action strategy set; $\beta$ (0 $\le \beta \le$ 1) is influence factor.

The GC seller GPMs make a joint action offer based on the green power trading price, generation efficiency, and environmental status such as cost recovery cycle and carbon asset. Therefore, a reinforcement learning algorithm is added to the set of trading strategies to improve the set of strategies selected by the participants’ actions. the set of strategies at moment *t* is denoted as $S_t$: $S_t \subseteq R$. The purchaser and seller of GCs choose their respective offer actions at a certain moment *t*.

The purchaser can separately compute the payment matrix $U_t$ of the purchaser for both parties under different strategy choices based on the defined payment function. It is difficult for buyers (QEs) to be informed of the state-action values Q of sellers (GPMs) to accurately find suitable strategies to cope with them, so we incorporate Q-learning methods to learn the action-state values of GPMs and adjust them to a targeted set of trading strategies.The game strategies of GPMs are learned through reinforcement learning to develop suitable bidding strategies for buyers.

The learning task of Step-T cumulative reward is added to the algorithm, starting from the initial state of the offerer, so that the offerer obtains an offer trajectory of GPMs with Step-T after learning:$\langle x_0, a_0, r_1, x_1, a_1, r_2,..., x_{t - 1}, a_{t - 1}, r_t, x_t \rangle$. In order to obtain the optimal strategy, the $\varepsilon -greedy$ algorithm is introduced, which selects one action uniformly at random from all actions with probability $\varepsilon$. The current optimal action is selected with probability 1 - $\varepsilon$, and the identified strategy is labeled as the “original strategy”. The policy that uses the $\varepsilon -greedy$ algorithm in the original policy is denoted as Eq. (7):

7 $$\begin{aligned} \pi (a|s) = {\left\{ \begin{array}{ll} 1-\varepsilon +\frac{\varepsilon }{|\mathcal {A}(s)|}, &{} \text{ if } a={\arg \max }_a Q^{\pi }(s,a)\\ \frac{\varepsilon }{|\mathcal {A}(s)|}, &{} \text{ if } a \ne {\arg \max }_a Q^{\pi }(s,a) \end{array}\right. } \end{aligned}$$

In GC trading process ,the sum of cumulative rewards for each pair of state-action Q in the trajectory is recorded as a one-time sampling value of cumulative rewards with respect to the GC seller. When multiple GC seller trajectories are obtained by sampling the GC seller multiple times, the cumulative reward sampling values obtained multiple times will be averaged using Eq. (8) to obtain an estimate of Q.

8 $$\begin{aligned} Q_n(k)=\frac{1}{n}((n-1)\times Q_{n-1}(k)+u_n)=Q_{n - 1} (k) + \frac{1}{n} (u_n - Q_{n - 1}(k)) \end{aligned}$$

During the Nash-Q intensive learning process for GC trading based on GPMs, QEs, and other participants, the corresponding iterative refinement of the Q-table is illustrated in Algorithm 1. To validate the effectiveness of Nash Q-Learning in optimizing GC trading and enhancing sustainability by promoting the increased use of renewable energy sources, a comprehensive experimental setup and an evaluation strategy are essential. We employ the trade-off between exploration and exploitation, known as the Exploration-Exploitation Tradeoff ($\epsilon$-greedy).

**Algorithm 1:**
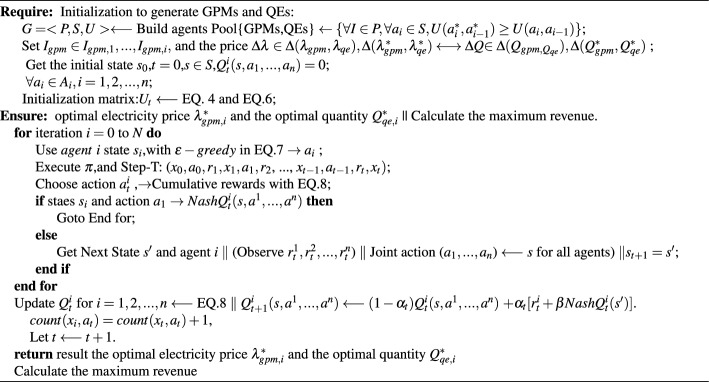
Collaborative algorithms based on game theory and Q-learning.

### Smart contracts and transaction execution

In this work,we integrate game models with smart contracts to solve the bargaining, consensus cooperation, and trading equilibrium problems in GC trading. Based on the Nash equilibrium strategy, a trading contract script under smart contract is established to dynamically implement the trading strategy (NES-SC).

The GC trading game model, coupled with the operational mechanics of smart contracts, guarantees that all transaction and management parties can dynamically and intelligently complete transactions within given constraints. To ensure a systematic approach to equilibrium and market clearing, the execution flow of this process is outlined in Algorithm 2.

**Algorithm 2:**
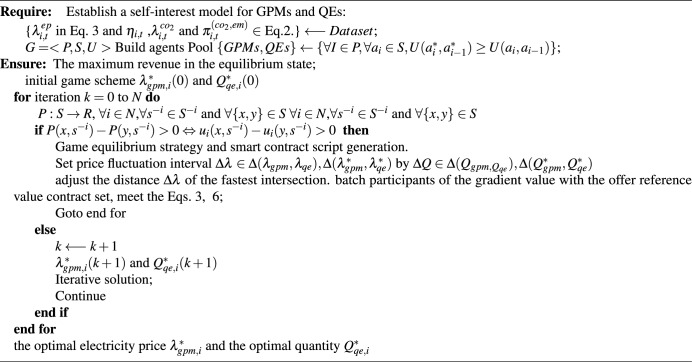
NES-GC Process Improvement for Smart Contracts.

## Results and analysis

### Basis of data

**Figure 3:**
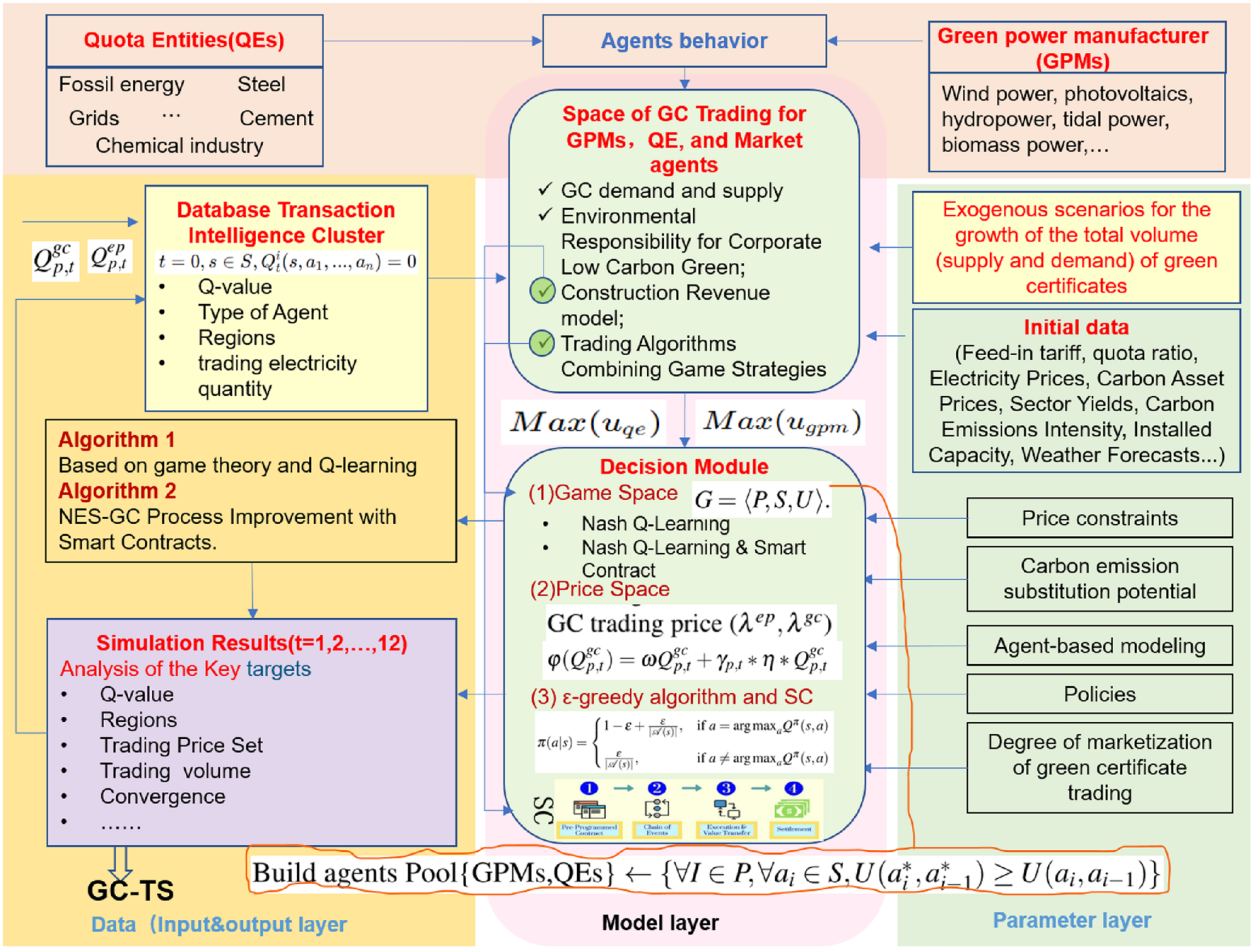
Methodological framework for logical relationships between models.

In this paper, we focus on the demand for Green Certificates (GCs) from enterprises in thermal power, chemical industry, iron and steel, and cement sectors within China’s eight major carbon emission categories. Three GC supply enterprises are selected to partake in the simulation transaction. The relationship between GC price, green power trading price, and carbon asset price is delineated as Eq. (5). The convergence and trading return behavior are governed by Eqs. (4), (6), with carbon emissions intensity data referenced in Table 1,and the methodological framework for logical relationships between models (Fig. 3).

This study simulates the Nash bargaining scheme for bilateral GC transactions, utilizing historical data of Wind-GC. CQ-fulfilling enterprises across different industries in 2020 are chosen as Qualified Entities (QEs) (see Table 1). In the case study, four QEs and three Green Power Markets (GPMs) (Wind) are selected to engage in the gaming process to substantiate the analysis. The algorithms 1, 2 are coded and executed in python3.9.

**Table 1:** Carbon emissions of CQ companies (2020, extracts from Chinese listed companies, CNY).

QEs	Enterprise attributes	Revenue (10 kt)	Carbon emissions (100 million CNY)	Carbon emission intensity (tons/10k CNY)	Data source
QE-1	Non ferrous	1860	8904	4.79	
QE-2	Steel	370	1758	4.75	$\star$
QE-3	cement	71	1430	20.06	*
QE-4	Comprehensive	4645	4337	0.93	
QE-5	Power plant	142	3694	25.98	$\star$
QE-6	Coal	338	3026	8.97	$\star$
QE-7	Petrifaction	21,060	17,094	0.81	
QE-8	Shipping	1713	2084	1.22	
QE-9	Communication	3038	1423	0.47	
QE-10	Chemical industry	167	831	4.98	

In addition, we take the base price as 0.43 (CNY)/kWh,the operating cost as 0.2 (CNY/kWh), $\omega$=0.96 (t./MWh) and combine the data in Table 1 (* is the estimated data of Zhong Chuang Carbon Investment & Finance magazine. Enterprises marked with $\star$ have their own disclosure, and enterprises marked with blank use their own public disclosure of data.) to verify the analysis.In this paper, we set $\lambda _{gpm,1}^{*}(0)=210,\lambda _{gpm,2}^{*}(0)=200,\lambda _{gpm,3}^{*}(0)=196$.

### Analysis of results

In the results analysis section, we present the performance of the GC-TS architecture and trading mechanism through simulations and experimental validation under varying market conditions and policy environments. By establishing a multi-agent reinforcement learning game equilibrium model and proposing a Nash Q-learning offer clearing mechanism, as well as integrating smart contracts to facilitate smart trading, we aim to validate the analysis of the convergence efficiency of the trading offer game, trading efficiency, and optimized trading benefits.

#### Convergence analysis

**Figure 4:**
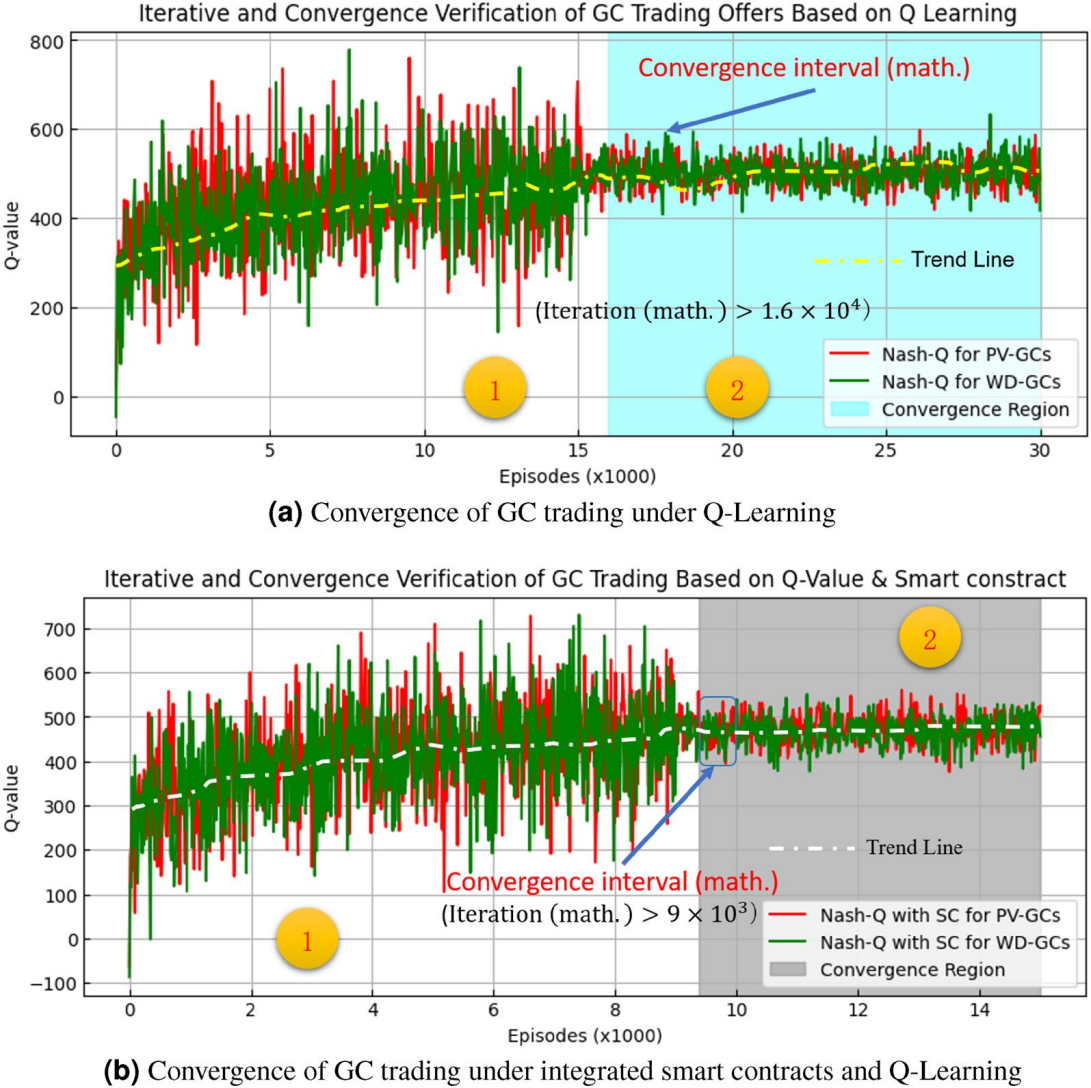
Convergence analysis of green certificate trading.

In Fig. 4, we observe the following:

1. On the Q-value Analysis aspect, Fig. 4a shows the Q-values of the Nash Q-learning algorithm for both photovoltaic (PV-GCs) and wind (WD-GCs) green certificates without the integration of smart contracts. The Q-values for photovoltaics tend to be slightly higher than those for wind, suggesting that photovoltaic projects might expect slightly higher returns in the simulated trading environment. In Fig. 4b, when smart contracts (SC) are introduced, the Nash Q-learning with SC demonstrates Q-values that stabilize at higher iterations with less fluctuation, indicating that smart contracts might offer additional stability to the trading process.
2. On the Convergence speed, Fig.4a indicates that the system begins to show convergence behavior after $1.6\times 10^4$ iterations. In contrast, Fig. 4b shows the onset of convergence earlier, after just $9\times 10^3$ iterations, suggesting that the integration of smart contracts may accelerate the convergence speed of the Nash Q-learning algorithm.
3. On consistency of GC trade clearing, both Fig. 4a and b, the stability of Q-values within the convergence interval signifies a consistent clearing process. This means that as learning progresses, trading strategies for both types of green certificates begin to align, with agents finding more optimal strategies for maximizing returns.

In summary, the analysis of these two Fig. 4a,b reveals the potential advantages of the Nash Q-learning model integrated with smart contracts in terms of convergence speed, stability, and consistency of trading strategies. These advantages may bring significant benefits to actual green certificate trading, thereby promoting the use of renewable energy and the overall development of the market.

#### Price analysis

**Figure 5:**
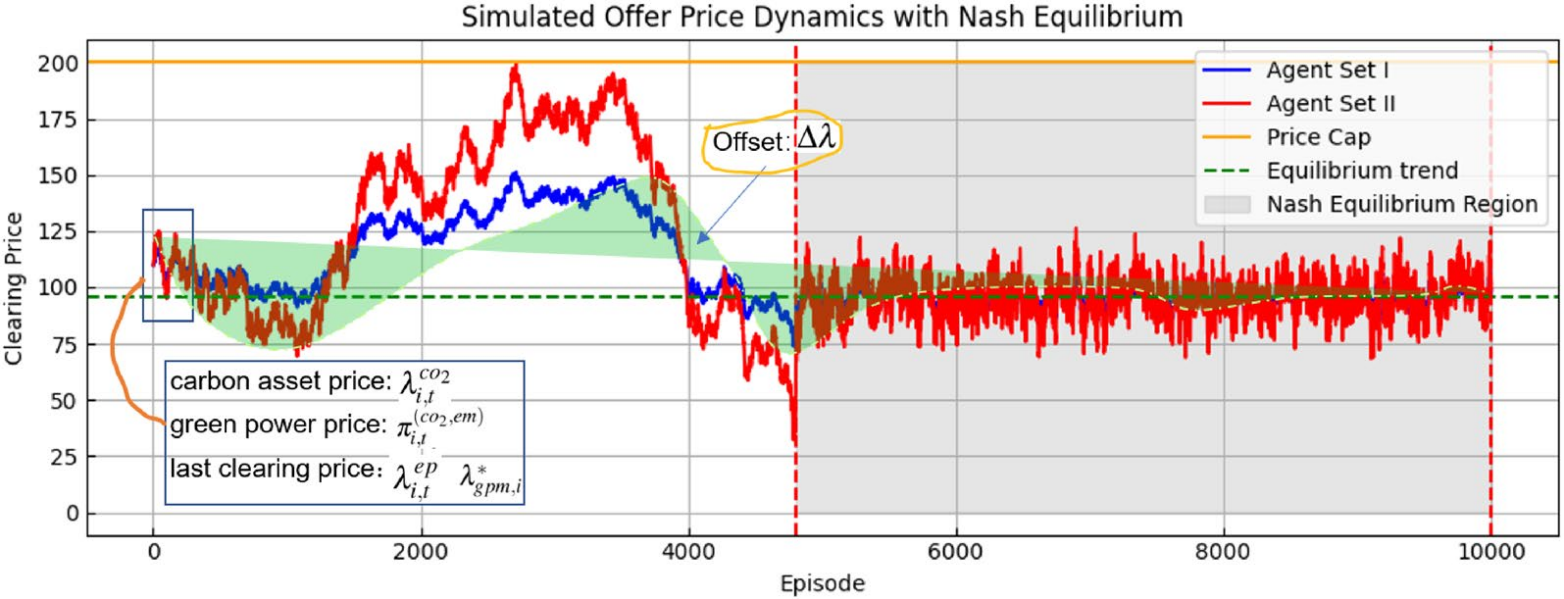
Trend analysis of GC price under the model learning process.

Figure 5 shows a simulation of how the bid prices of the two players change over the episode. The Nash equilibrium is represented by the green solid line, with the Episode outside the 5000 range and bids converging to the value of the Nash equilibrium. The chart displays the evolving dynamics of offer prices from two groups of agents involved in the green certificate market, categorized as Agent Set I and Agent Set II, within a Nash Q-learning framework. Their pricing strategies are informed by the current green power price($\pi _{(co_2,em)}$), carbon asset trading price ($\lambda _{i,t}^{co_2}$), and the last round’s clearing price($\lambda _{i,t}^{ep}$,$\lambda _{(gpm,i)}^*$). Regarding the offer trend and adjustment (Offset $\delta \lambda$), the green power price and carbon asset price provide the base price parameters for the trading parties. As seen in Fig. 5, these prices generally fall within the range of offers from both sides; the offer adjustment (Offset $\delta \lambda$) indicates that both sides continually adjust their offers in search of market equilibrium. Before reaching consistency within the Nash Equilibrium Region, there might be a significant difference in the offers from the buyer and seller. The equilibrium trend analysis shows that (1) after initial fluctuations, the offers from both trading parties begin to converge toward the equilibrium trend line (dashed green line), indicating the market is moving towards stability and consistency; (2) in the chart, around the 2000 to 4000 episode range, offers begin to converge within the Nash Equilibrium Region, suggesting that after a series of dynamic adjustments, the market has found an equilibrium price acceptable to both sides. Additionally, the analysis of convergence and market clearing in the chart found that as the number of episodes increases, the offers from Agent Set I and Agent Set II tend to stabilize within the Nash Equilibrium Region. This convergence demonstrates the effectiveness of the proposed Nash Q-learning offer clearing mechanism, capable of implementing smart trading by integrating smart contracts, thus enhancing trading efficiency and optimizing trading benefits.

#### Comparison of two algorithms

**Figure 6:**
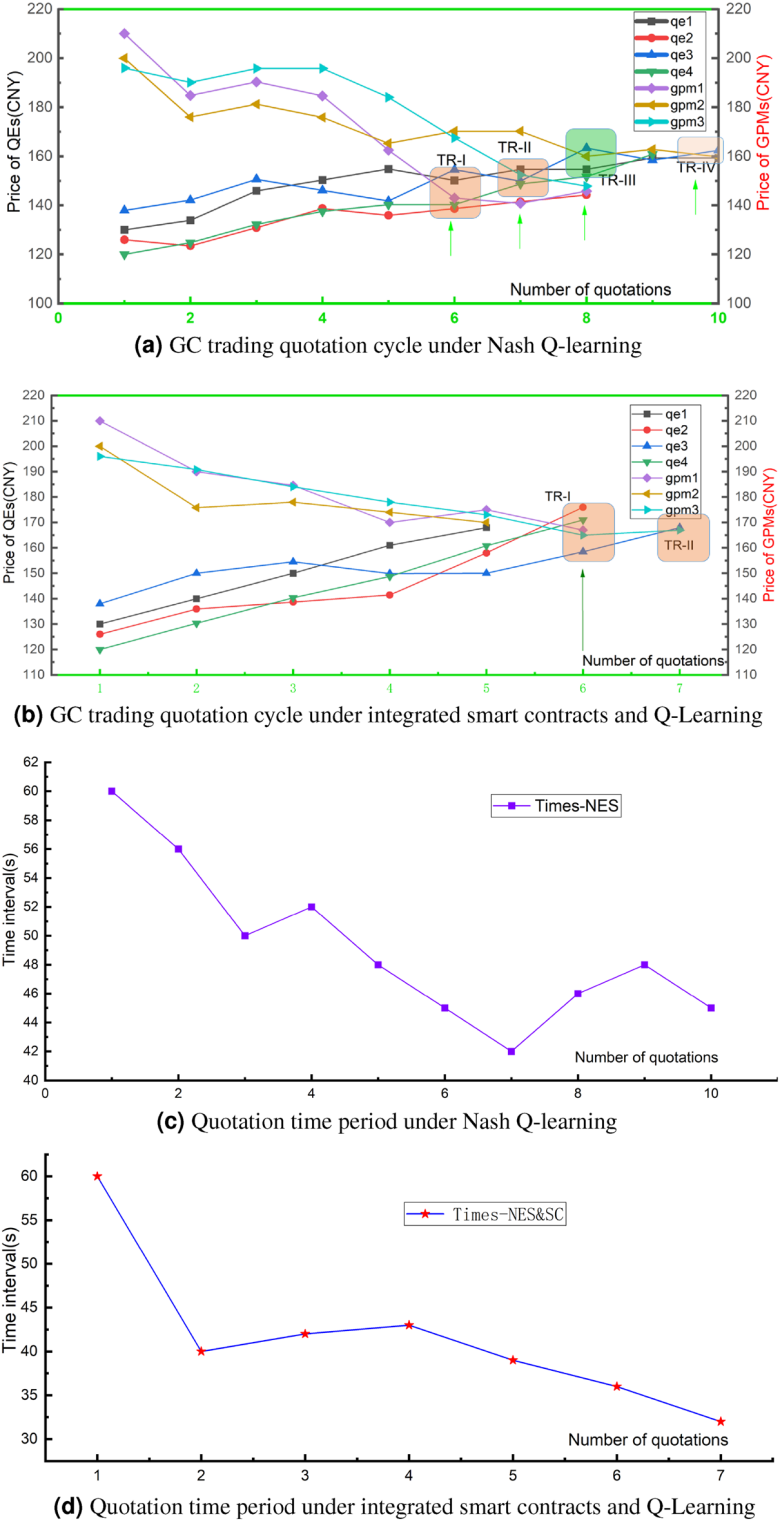
Quotation and time period in two models (Nash Q Learning: algorithm1, Nash Q Lerning &SC: algorithm 2) in case.

**Figure 7:**
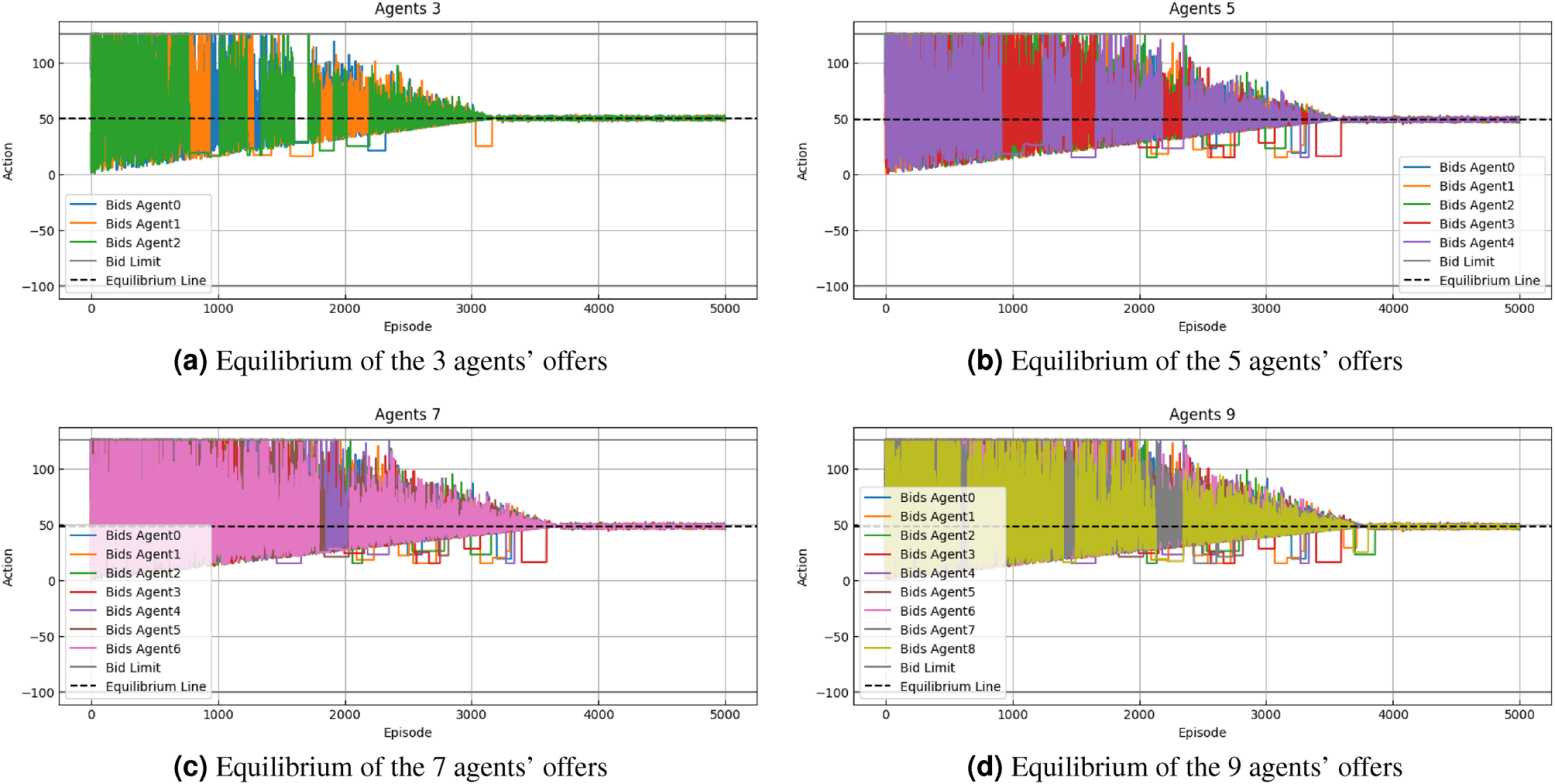
Robustness analysis of green certificate trading models.

Using the algorithm 1,2, we compare the speed of convergence after 5000 iterations of 100 3-agent simulations, 20 5-agent simulations, and 20 7-agent simulations. The game convergence scenario becomes more difficult to converge as the number of intelligences increases. This is illustrated by the example runs with 3, 5, 7, and 9 trading intelligence in Figs.6, 7.

In the Nash Q-learning (NES) and Nash Q-learning & Smart Contract (NES-SC) model model validation cases, a total of seven intelligences, QEs, and GPMs, were used to participate in the GC quotation trading. Four QEs and three GPMs achieve 10 rounds of quotes in this case. Figure 6a,c are the quotation and strategy spending time in NES mode, respectively. The supply and demand sides continuously adjust the price, trading volume, and other game strategies, and multiple quotes form a trading trend when $k\ge 5$. There are TR-I, TR-II, TR-III, and TR-IV transaction clusters that satisfy the price and volume demands in the offer cycle in Fig.6a, and the model cannot guarantee that all demands are satisfied when $k = 10$ and the transaction is not yet completed. The number of offers increases, the amount of information obtained increases, and the time spent by the strategy subsequently decreases as in Fig.6c. In the same data case, we performed a case study of NES-SC, and Fig. 6b,d are the offer and strategy spend time, respectively. Figure 6b shows that both sides basically complete the transaction (two transaction clusters TR-I and TR-II) when $k=7$. Compared with Fig. 6a,c, the transaction success rate of both sides is improved and the strategy spending time is shortened. In conclusion, the two methods are consistent in the trend of quotes, and the NES-SC method is based on the NES model, so both methods can effectively support the trading platform, and NES-SC has better advantages in transaction efficiency.

#### Robustness analysis

To validate the robustness of the green certificate trading model, we increased the complexity of transactions by expanding the number of agents involved. The study employs cluster agents representing different industries, such as power grid, steel, chemical, and thermal power, with each group consisting of several individual entities participating in collective quotation trading.

In Fig. 7, we observe the bidding behavior of agent groups with 3, 5, 7, and 9 agents participating in Nash Q-learning and integrated smart contract trading strategies. These charts reflect how agents’ bidding actions change over time and attempt to approach the equilibrium line. In Fig. 7a, the bidding behavior of the agents exhibits significant fluctuations, which may reflect the uncertainty of decision-making in a smaller group of agents. Despite the fluctuations, bids tend to converge toward the equilibrium line over time.As the number of agents increases, the volatility of the bidding behavior seems to increase, indicating that reaching a consensus becomes more difficult in a larger group. However, over time, bids tend to stabilize and move closer to the equilibrium line (in Fig. 7b–d). NeverthelessIn,from the Fig. 7c,d, the largest group of agents, despite significant volatility, the bidding trends gradually approach the equilibrium line over the long term.So this indicates that even in more complex multi-agent environments, the combined strategy of Q-learning and smart contracts can still guide groups of agents toward collaboration and equilibrium.

From these simulations, we can conclude that as the number of agents increases, the complexity of the trading strategy and the difficulty of reaching equilibrium also increase correspondingly. The integration of Nash Q-learning and smart contracts is crucial for enhancing the coordination of group behaviors and driving toward an equilibrium in trades, especially in complex trading environments involving more agents. These simulations emphasize the importance of considering agent diversity and collective behavior patterns when designing green certificate trading systems.

### Comparison of green certificate trading

As the actual value of GCs in the future carbon neutrality is more and more emphasized by the Chinese government, and the value-added benefits of carbon quota enterprises through the carbon offset mechanism of GCs are taken into account.

**Figure 8:**
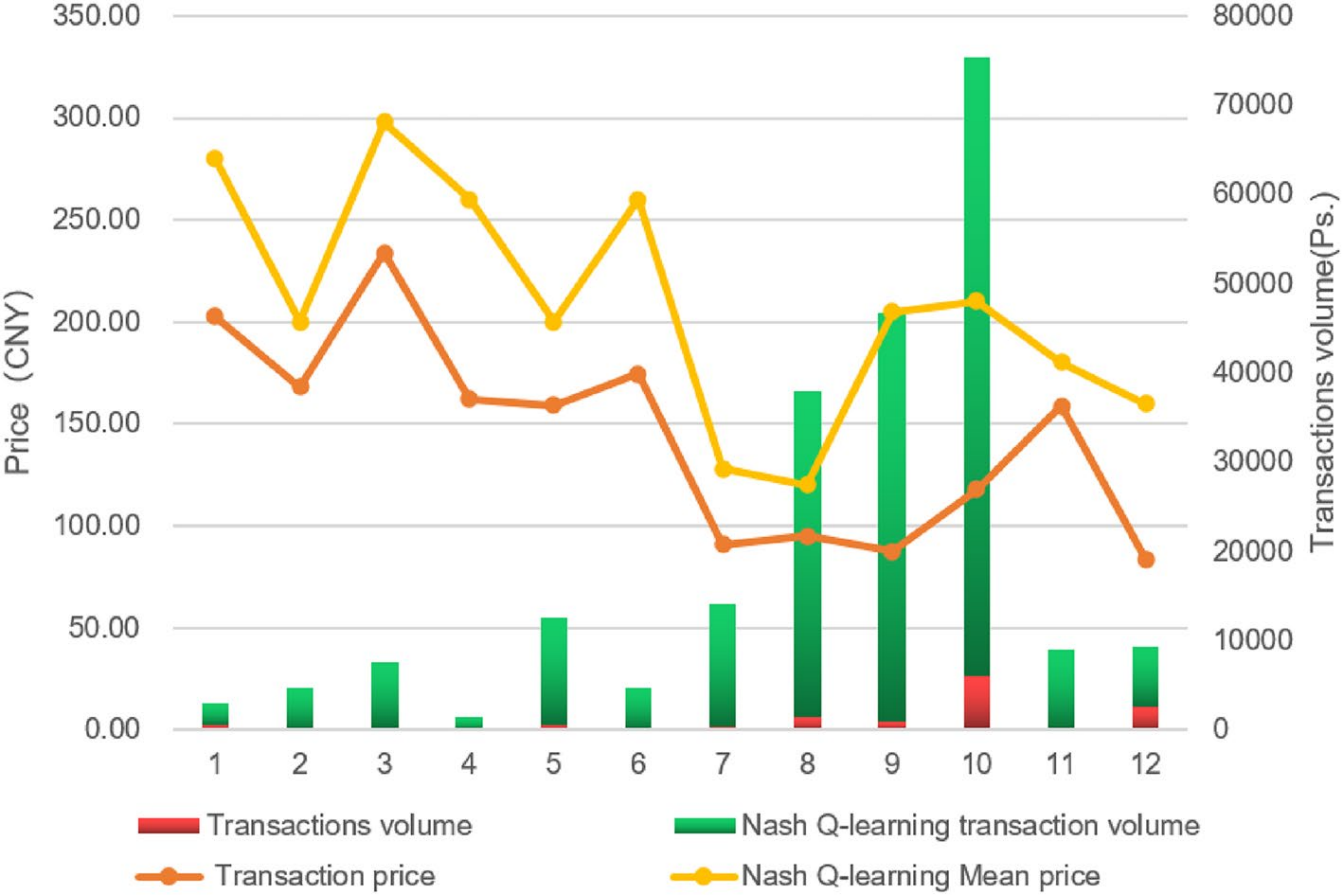
Comparison of price and volume based on Nash Q-Learning simulation with actual transactions.

Figure 8 is the analysis of the monthly mean values of the quoted price and trading volume under the gaming strategy of the participating counterparties. The trading model designed in this paper underpins the overall transaction price and volume. Using an equilibrium strategy, it swiftly locates the trading point for each participant, and compared with the trading data from 2021, the transaction volume has increased by up to 30 times. The average transaction price induced by the Nash Q-learning strategy (yellow line) is generally higher than the actual transaction price (orange line), thanks to the game-theoretic strategy negotiation and bid adjustments of the Nash Q-learning model.

In terms of transaction volume, the graph shows a significant peak in Nash Q-learning transaction volume (green bar) in the ninth month, which occurs concurrently with the peak of the Nash Q-learning mean price (yellow line). The data reveals that the forecasted transaction volume by Nash Q-learning reached nearly 70,000 units, whereas the actual volume was only about 2000 units. As for pricing, the predicted price by Nash Q-learning for the ninth month reached close to 300 CNY, while the actual price was around 200 CNY. The actual transaction volume (red bar) and price (orange line) exhibit some volatility with noticeable peaks and valleys. The transaction volume prediction by Nash Q-learning (green bar) is more stable.

The Fig. 8 illustrates the potential value-added benefits of applying the Nash Q-learning strategy in the GC market, reflecting its capability to quickly locate optimal trading points for participants, potentially leading to an increase in price and volume compared to historical data. This aligns with the objective of enhancing the effectiveness of the GC trading model and the carbon offset mechanism in the context of carbon neutrality goals.

## Conclusion

Given the complexity of issuing, verifying, and trading GCs in China, coupled with the challenges brought forth by policy shifts, it is imperative to ensure that the market-based GC trading system is bolstered by adequate mechanisms and technological support. This study proposes an architecture for a zero-carbon energy Green Certificate Trading System (GC-TS) that leverages an equilibrium strategy, enhancing the efficiency of GC trading quotes and facilitating multi-party collaboration through the incorporation of Q-learning, smart contracts, and an effectively integrated multi-agent Nash strategy. Initially, the system integrates GC trading with electricity and carbon asset markets, formulating pricing strategies across these domains. Subsequently, a certificate-electricity-carbon efficiency model is designed to maintain consistency across the GC, green electricity, and carbon markets. Aiming for diversified GC trading, a multi-agent reinforcement learning game equilibrium model is established, alongside a Nash Q-learning offer clearing mechanism that employs smart contracts for intelligent trading, thereby increasing the convergence efficiency of the trading offer game and enhancing trading efficacy while optimizing benefits for all parties involved.

In summary, this research plays a constructive role in advancing the development of renewable energy in China. It highlights the scalability of the GC-TS and its potential as a reference model for future GC trading platforms in China. The system offers a swift avenue for green power producers to subsidize the costs of renewable energy generation through GC proceeds, facilitating the involvement of carbon quota enterprises in carbon emission responsibility compliance, and promoting the growth of renewable energy sources.

## Data Availability

The datasets used and/or analyzed during the current study are available from the corresponding author upon reasonable request.
