# Author Correction: Enhancing renewable energy certificate transactions through reinforcement learning and smart contracts integration

**DOI:** 10.1038/s41598-025-89513-z

**Published:** 2025-02-13

**Authors:** Qingsu He, Jinsong Wang, Ruijie Shi, Yifan He, Muqing Wu

**Affiliations:** 1 State Grid Digital Technology Holdings Co., Ltd. (State Grid Xiongan Financial Technology Group Co., Ltd.), Beijing, 100010 China; 2 Case Western Reserve University Cleveland, Cleveland, 44106 USA; 3 University of California, Santa Cruz, 95064 USA; 4 College of Information and Communication Engineering, Beijing University of Posts and Telecommunications, Beijing, 100876 China

Correction to: *Scientific Reports* 10.1038/s41598-024-60527-3, published online 12 May 2024

The original version of this Article contained an error in the spelling of the author Jinsong Wang, which was incorrectly given as Jingsong Wang.

The original Article has been corrected.

